# FGFR1 promotes the stem cell-like phenotype of FGFR1-amplified non-small cell lung cancer cells through the Hedgehog pathway

**DOI:** 10.18632/oncotarget.7701

**Published:** 2016-02-25

**Authors:** Wenxiang Ji, Yongfeng Yu, Ziming Li, Guan Wang, Fan Li, Weiliang Xia, Shun Lu

**Affiliations:** ^1^ Shanghai Lung Cancer Center, Shanghai Chest Hospital, Shanghai Jiao Tong University, Shanghai 200030, China; ^2^ State Key Laboratory of Oncogenes and Related Genes, Renji-Med X Clinical Stem Cell Research Center, Ren Ji Hospital, School of Biomedical Engineering, Shanghai Jiao Tong University, Shanghai 200030, China; ^3^ Genomics Center, WuXiAppTec Co., Ltd., Shanghai 200131, China

**Keywords:** FGFR1, GLI2, lung squamous cell cancer, stem cell-like phenotype

## Abstract

Cancer stem cell-like phenotype is critical for tumor formation and treatment resistance. *FGFR1* is found to be amplified in non-small cell lung cancer, particularly in the lung squamous cell cancer (LSCC). Whether FGFR1 contributes to the maintenance of stem cell-like phenotype of FGFR1-amplified lung cancer cells remains elusive. In this study, treatment with FGFR1 inhibitor AZD4547 suppressed the growth of tumor spheres and reduced ALDH positive proportion in FGFR1-amplified lung cancer cells in vitro, as well as inhibited the growth of oncospheres and parental cells in xenograft models. Knockdown of FGFR1 recaptured the similar effect as AZD4547 in vitro. Furthermore, activation of FGFR1 and subsequently its downstream ERK signaling enhanced the expression and transcriptional activity of GLI2, which could be blocked by FGFR1 inhibitor/silencing or ERK inhibitor. Knockdown of GLI2 directly inhibited the stem-like phenotype of FGFR1-amilified cells, whereas overexpression of GLI2 sufficiently rescued the phenotype caused by FGFR1 knockdown. Notably we also identified a correlation between FGFR1 and GLI2 expressions from clinical data, as well as an inverse relationship with progression free survival (PFS). Together our study suggests that the FGFR1/GLI2 axis promotes the lung cancer stem cell-like phenotype. These results support a rational strategy of combination of FGFR1 and GLI inhibitors for treatment of FGFR1-amplified lung cancers, especially LSCC.

## INTRODUCTION

Lung cancer is the leading cause of cancer related death worldwide with a mere 16.8% 5-year survival rate after diagnosis [1]. The emergence of targeted therapy such as EGFR inhibitors and anaplastic lymphoma kinase (ALK) inhibitors have significantly changed the treatment strategy against lung adenocarcinoma AC, leading to marked improvements in patient survival. Compared with AC, the mainstream treatment regimens for lung SCC (LSCC) are still platinum-based chemotherapeutics, and the advance of targeted therapy in LSCC is rare. Thus, it is essential to further explore the molecular mechanism underlying the occurrence and development of lung cancer, especially in LSCC, and find out the druggable driver genes for the future intervention strategies.

Increasing data have demonstrated that NSCLC contain a small group of stem cell-like cells (cancer stem-like cells, CSCs) [2, 3]. CSCs are tumor cells with enhanced ability for tumor generation. They are capable of dividing asymmetrically to produce one progeny that maintains the stem cell capacity for self-renewal, and the other that will differentiate and produce tumor-constitutive, phenotypically diverse cancer cells [4]. These highly tumorigenic cells maintain self-renewal by activating several classical developmental pathways including Wnt, Hedgehog (Hh), and Notch. Moreover, several stem cell-related genes such as SOX2 [5], OCT3/4[6], and NANOG [6] also participate in this process. The existence of CSCs has been considered as a source of drug resistance and recurrence, making them a likely source of therapeutic failure [2]. Previously, ALDH-positive cells had been proposed to exhibit cancer stem cell-like properties, which were used to assay the proportion of cells with stem cell-like properties in cancer cells [7, 8].

FGFR1 amplification is a prominent gene alteration in NSCLC, especially in LSCC. FGFR1 amplification was found in 10–22% of LSCC but in only 0–5.2% of lung AC [9-12]. FGFR1 has been considered as a promising therapy target in FGFR1-amplified tumors [11, 13-15]. However the role of FGFR1 pathway in maintaining the stemness phenotype of CSCs is complicated. It was reported that FGFR1 activation could lead to the differentiation of granule cell precursors (GCPs) [16]. On the contrary, the upregulation of FGFR1 could promote stem/progenitor cell-like properties in breast carcinomas [17]. However, the role of FGFR1 in maintaining the stem cell-like phenotype of LSCC is still elusive.

Herein, for the first time we demonstrated that FGFR1 maintained a highly tumorigenic phenotype in lung cancer cells harboring FGFR1 amplification, and in LSCC tumors. Our work revealed the mechanism, preliminary, through which FGFR1 regulated the stem cell-like phenotype of LSCC.

## RESULTS

### Inhibition of FGFR1 represses ALDH activity and oncosphere formation in NSCLC cells *in vitro* and *in vivo*

**Figure 1:**
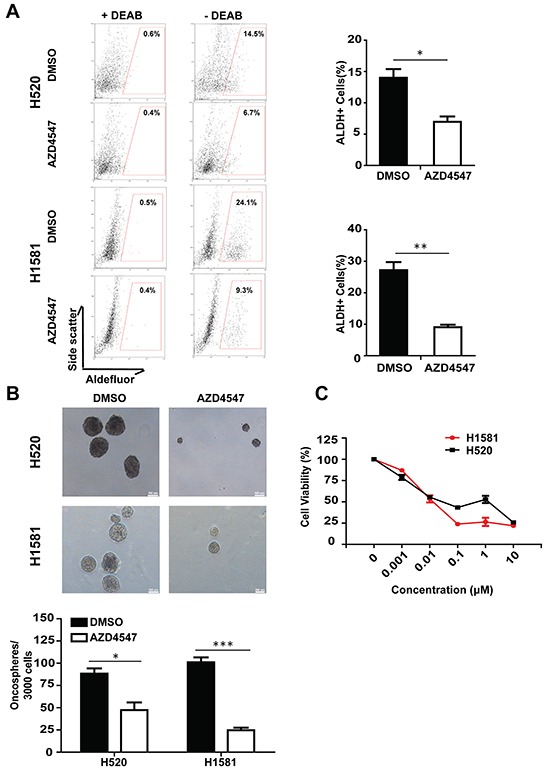
Inhibition of FGFR1 activity suppressed stem cell traits in NSCLC *in vitro* **A.** ALDH activity assay in NSCLC cell lines H520 and H1581. Representative flow cytometry data were shown for each cell line, indicating the proportion of aldefluor positive (% ALDH1^+^) cells after treatment with FGFR1 inhibitor AZD4547 (1 μM) for 5 days. Experiments were repeated three times and results were summarized in the bar graph (Error bars represent SEM, N=3;*p<0.05 and **p<0.01, Student's t test). **B.** Oncosphere formation assay in NSCLC cell lines H520 and H1581. The representive photographs of oncospheres were shown at low magnifications, with the scale bar representing 100μm. The number of formed oncospheres per 3000 cells plated was summarized, with or without the treatment of AZD4547 (1 μM). (Error bars represent SEM, N=6; *p<0.05, **p<0.01 and ***p<0.001, Student's t test). **C.** The cytotoxicity of FGFR1 inhibitor against the oncosphere cells. The oncosphere cells of H520 and H1581 were treated with FGFR1 inhibitor AZD4547 with various concentrations for 96h. Results were expressed as percentage of DMSO control (Error bars represent SD, N=6).

To test if FGFR1 signaling participates in the stem cell-like properties of NSCLC cells, we first explored the relationship between FGFR1 and ALDH activity. FGFR1-amplified H520 and H1581 cells were treated with DMSO or AZD4547 (1 μM) (an FGFR1 inhibitor) in low serum medium. The medium was changed every day. After 5 days, equal number of cells were collected and subjected to ALDH activity analysis. As shown in Figure 1A, inhibition of FGFR1 with AZD4547 decreased the fraction of ALDH-positive cells dramatically both in H520 (p<0.05) and H1581 (p<0.01) cells. Compared with their respective vehicle controls, the percentages of ALDH-positive cells declined from 14.5% to 6.7% in H520 cells and from 24.1% to 9.3% in H1581 cells. Since AZD4547 was a multitargeted FGFRs kinase inhibitor, we corroborated the effect of this inhibitor by experiments using FGFR1 silencing. Knockdown of FGFR1 led to almost complete loss of FGFR1 protein (Supplementary Figure S1A). As shown in Supplementary Figure S1C, compared with its vector control, FGFR1 KD led to a significant decrease of ALDH-positive proportion, from 14.6% to 2.8% in H520 (p<0.01) and 49.6% to 26.3% in H1581 (p<0.01).

Sphere assays are routinely applied to measure the potential of cells to show stem cell traits once they are removed from their original microenvironmental niche [18]. Corroborated with the ALDH activity analysis, we found that FGFR1 inhibitor could suppress the growth of H1581 and H520 oncospheres (Figure 1B). Moreover, AZD4547 inhibited the oncosphere viability of H1581 and H520 cells. The concentration at which AZD4547 was capable of suppressing the oncosphere viability by 50% was estimated in H520 (IC_50_= 0.12 μM) and H1581 (IC_50_=0.023 μM) cells (Figure 1C). Likewise, FGFR1 knockdown significantly suppressed sphere-forming ability and *in vitro* self-renewal capacity of H520 and H1581 cells (Supplementary Figure S1B), and retarded the growth of H520 and H1581 oncospheres (Supplementary Figure S1D).

To further explore the effect of AZD4547 against oncospheres and parental cells (Supplementary Figure S2A) *in vivo*, we used H1581 as a model. Compared with the vehicle control group, treatment with AZD4547 (12.5 mg/kg/d) could effectively slowed tumor growth both in mice engrafted with oncospheres and in those with parental cells (Supplementary Figure S2B and S2C). At the end of the experiments, the tumors were excised. Pooled tumors were dissociated and analyzed for percentage of ALDH positive cells. In the cells from parental xenograft, the ALDH positive cells were 21.6% (AZD4547) vs. 40.1% (vehicle), whereas in the oncosphere cells, the ratio was 58.3% (AZD4547) vs. 83.9% (vehicle) (Supplementary Figure S2D).

Thus, the similar results of FGFR1 inhibitor and FGFR1 KD indicated that interference with FGFR1 function inhibited ALDH activity and oncosphere growth in FGFR1-amplified cells.

### Activation of FGFR1 stimulates the expression of stem cell markers

**Figure 2:**
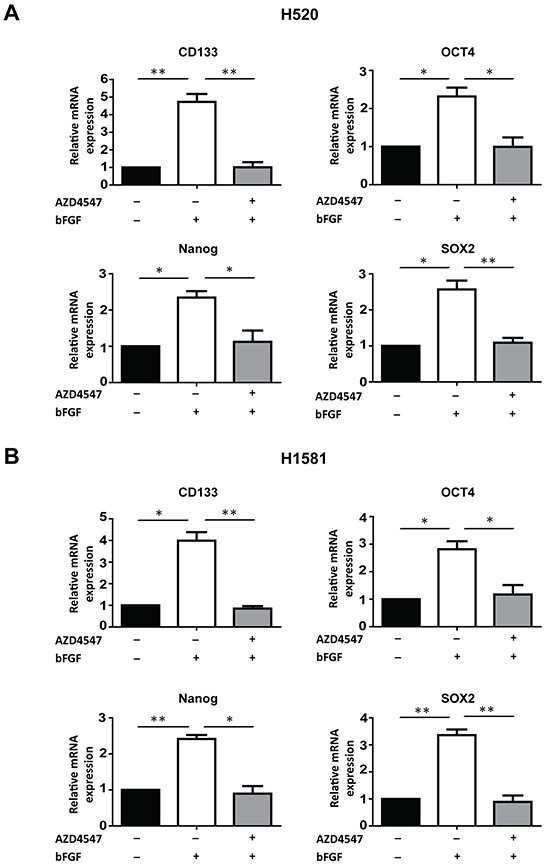
FGFR1 regulates the expression of stem cell markers The mRNA levels of stem cell markers CD133, NANOG, OCT4 and SOX2 inH520 **A.** and H1581 **B.** cells under untreated, bFGF-treated, and bFGF plus AZD4547-treated conditions were determined by real-time quantitative PCR (Error bars represent SEM, N=3;* p<0.05 and ** p<0.01, Student's t test).

To further explore the relationship between FGFR1 signaling and cancer stem cell traits, we measured the mRNA levels of stem cells markers CD133 [8], NANOG [6], OCT4 [6] and SOX2 [5], when FGFR1 signals were activated by bFGF, and in the presence of FGFR1 inhibitor. Serum-deprived H520 and H1581 cells were treated by bFGF (20 ng/ml) and heparin (10 μg/ml) for 24h with or without AZD4547 (1 μM). Upon stimulation by bFGF, expressions of stem cell markers CD133, NANOG, OCT4 and SOX2 increased 2-4 fold both in H520 and H1581 cells, and this stimulation could be fully blocked by AZD4547 (Figure 2A and 2B). These results provided further evidence that FGFR1 signaling could be involved in the regulation of cancer stem cells in FGFR1-amplified lung cancer cells.

### bFGF/FGFR1 activates ERK phosphorylation and upregulates the expression of GLI2 and its transcriptional activity

**Figure 3:**
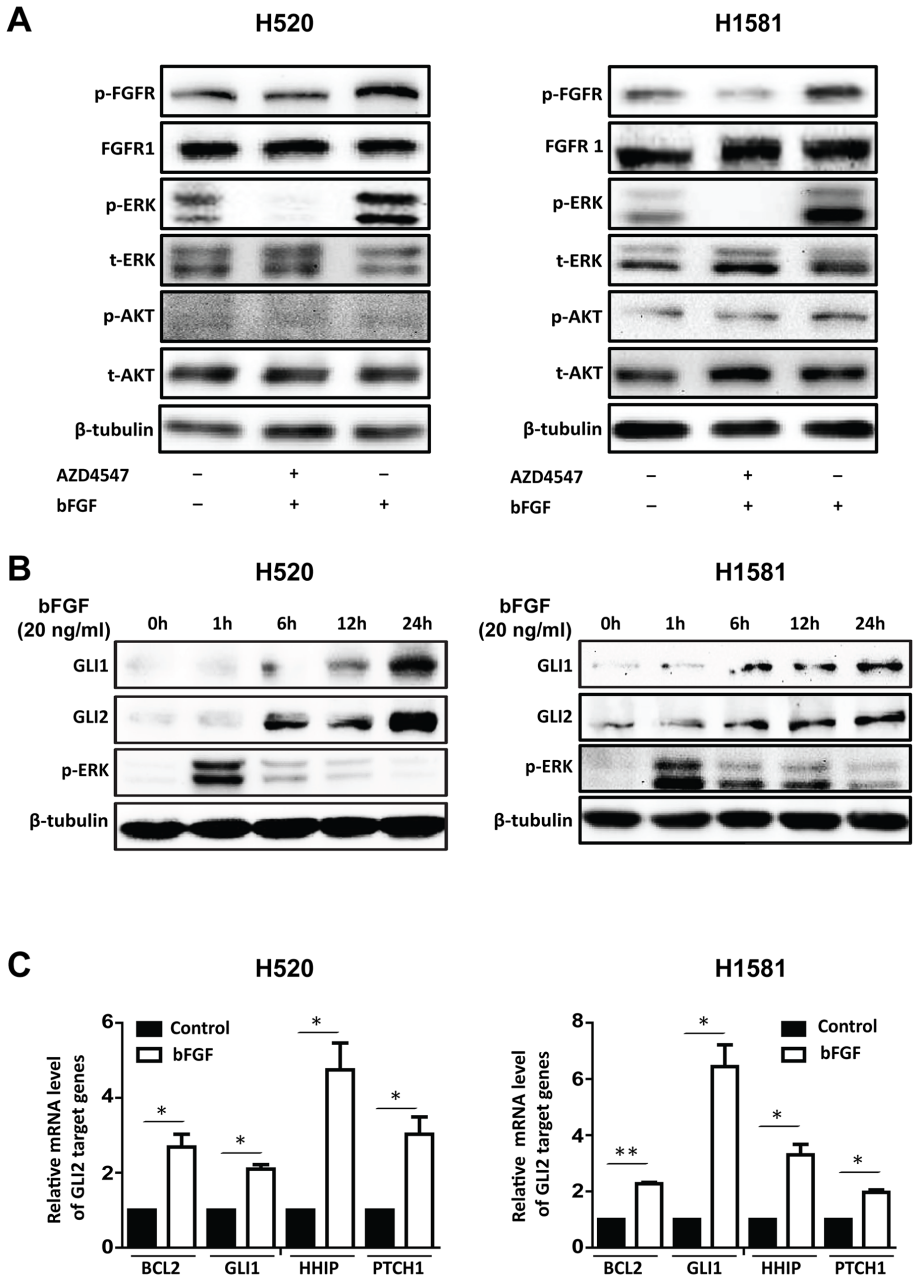
bFGF/FGFR1 activates ERK phosphorylation and upregulates the expression of GLI2 and its transcriptional activity **A.** Western blot assay FGFR1 activation and its downstream kinase activity. H520 and H1581 cells were stimulated with bFGF (20 ng/ml) and heparin (10 μg/ml) for 20 min with or without pretreatment ofAZD4547 (1 μM) for 40min. Cells were analyzed for phospho-FGFR (p-FGFR), FGFR1, phospho-ERK (p-ERK), total ERK (t-ERK), phospho-AKT (p-AKT) and total AKT (t-AKT). β-tubulin served as loading control. **B.** bFGF stimulates the expression of GLI1 and GLI2 in H520 and H1581 in a time-dependent manner. Cells were treated with bFGF (20 ng/ml) plus heparin (10 μg/ml) for different durations and assayed for GLI1, GLI2 and p-ERK levels by Western blot. **C.** The expression of GLI2 target genes BCL2, GLI1, HHIP and PTCH1 in H520 and H1581 cells with or without bFGF (20 ng/ml) plus heparin (10 μg/ml) treatment for 24 hours were examined by real-time PCR (Error bars represent SEM; N=3, *p<0.05 and **p<0.01, Student's t test).

To figure out the underlying mechanism through which, FGFR1 regulates the cancer stem cell properties in FGFR1-amplified lung cancer cells. First we tested the phosphorylation status of the downstream molecules following the activation of FGFR1. H520 and H1581 cell lines were stimulated by bFGF (20 ng/ml) plus heparin (10 μg/ml) for 20 min, with or without the pretreatment of AZD4547 (1 μM) for 40min. Then the cells were collected and analyzed by Westernblot. We found that both H520 and H1581 cells had a relatively high basal activation of FGFR1 pathway, including FGFR1 and its downstream component ERK, but the phosphorylation of FGFR1 and ERK could be significantly further activated by bFGF (Figure 3A). Such activation could be blocked by the pretreatment of AZD4547 (1 μM). Notably, the AKT phosphorylation (another main downstream pathway of FGFR) apparently was not influenced by bFGF or AZD4547, as described in previous studies [13, 15, 19] (Figure 3A).

Hedgehog signaling pathway plays an important role in maintaining the stemness of LSCC [20]. We hypothesized that FGFR1 might effect in stem cell traits through the regulation of the Hedgehog pathway. As GLI2 is a key transcriptional factor of the Hedgehog pathway and is reported to be activated predominantly in LSCC, especially the classical subtype [18, 21-23], we then tested if activation of FGFR1 could affect GLI2. We found that bFGF stimulated the phosphorylation of ERK and the expression of GLI1 and GLI2 (Figure 3B). The ERK phosphorylation occurred immediately after bFGF stimulation followed by the upregulation of GLI1 and GLI2 in a time-dependent manner (Figure 3B). As a crucial transcriptional factor in Hedgehog pathway, GLI2 works by driving the expression of its target genes, such as BCL2 [24], GLI1 [25], HHIP [24, 26], and PTCH1 [25]. In our work, we found that after bFGF treatment for 24h, the mRNA level of these four GLI2 target genes increased 2-6 folds in H520 and H1581 cells (Figure 3C). Together, these results indicated that bFGF/FGFR1 signaling activated ERK pathway and induced the expressions of GLI2 and its target genes.

### FGFR1 stimulates GLI2 expression through ERK pathway but not through the classical Sonic Hedgehog pathway

**Figure 4:**
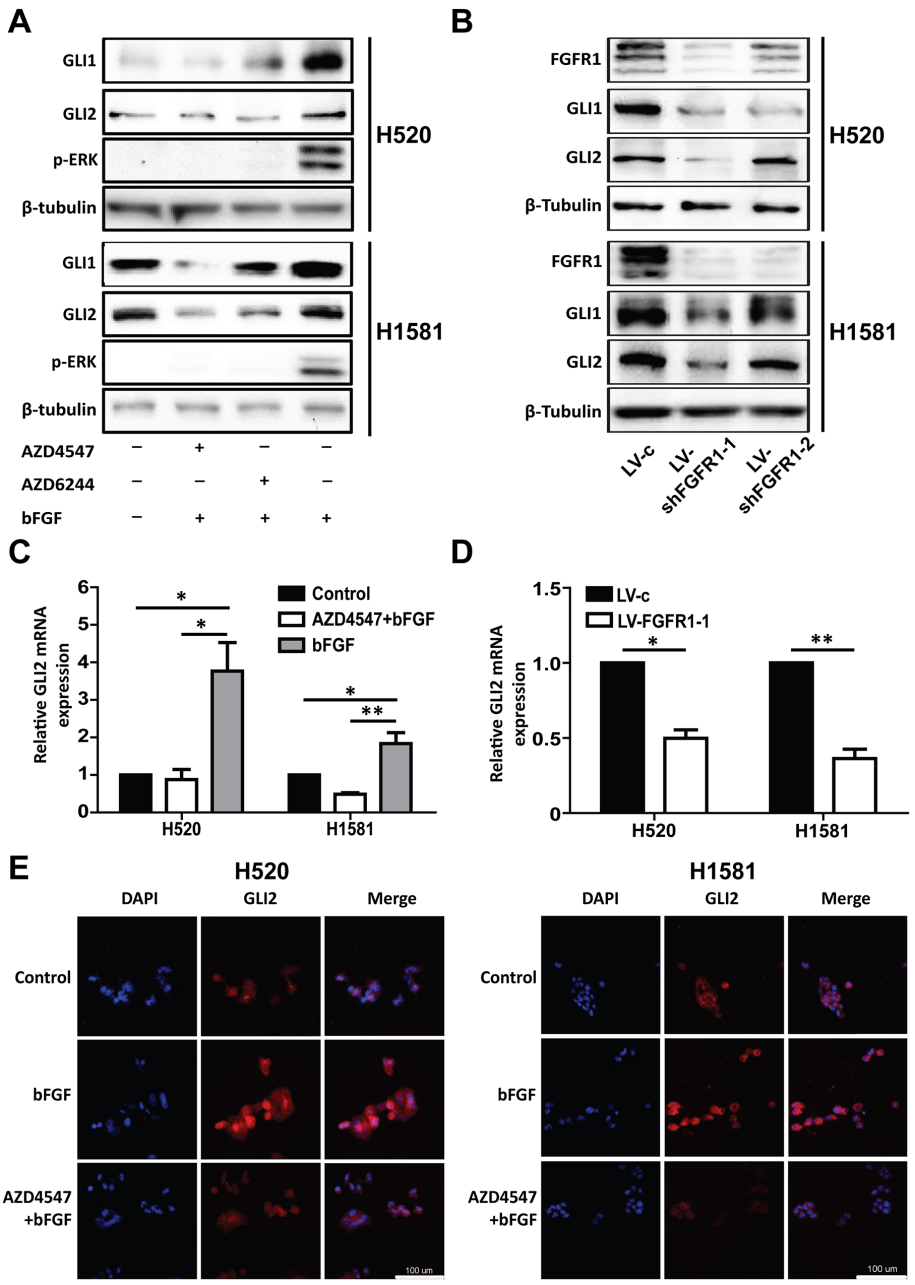
Inhibition ofFGFR1 suppresses the expression of GLI2 **A.** Western blot analysis of the protein level of GLI1 and GLI2 and phosphorylation of ERK in H520 and H1581 cells after treatment with bFGF (20 ng/ml) plus heparin (10 μg/ml)in combinations of FGFR1 inhibitor AZD4547 (1 μM) or MEK/ERK inhibitor AZD6244 (1 μM). **B.** Western blot analysis shows GLI1 and GLI2 expression levels in H520 and H1581 cells that were infected with control lentivirus (LV-c) or shRNA targeting FGFR1 (LV-shFGFR1-1 and LV-shFGFR1-2). **C.** The relative mRNA level of GLI2 in H520 and H1581 cells after treatment with bFGF (20 ng/ml) plus heparin (10 μg/ml) with or without AZD4547 (1 μM). (Error bars represent SEM; N=3, *p<0.05 and **p<0.01, Student's t test). **D.** The relative mRNA level of GLI2 in H520 and H1581 cells that were infected with control lentivirus (LV-c) or shRNA targeting FGFR1 (LV-shFGFR1-1) (Error bars represent SEM; N=3, *p<0.05 and **p<0.01, Student's t test). **E.** Immunofluorescence microscopic analysis of GLI2 in H520 and H1581 cells treated with bFGF (20 ng/ml) plus heparin (10 μg/ml) or co-treated with bFGF (20 ng/ml) plus heparin (10 μg/ml) and AZD4547 (1 μM). DAPI was used to stain nuclei. Scale bar = 100 μm.

To further test if there was a direct link between ERK phosphorylation and GLI2 expression, we included ERK inhibitor AZD6244 [27] in the following experiments. H520 and H1581 cells were activated similarly by bFGF (20 ng/ml) plus heparin (10 μg/ml) for 24h, with pretreatment of AZD4547 (1 μM) or AZD6244 (1 μM) to block the FGFR1 or the MEK/ERK pathways, respectively. The ERK inhibitor AZD6244 could block the ERK phosphorylation and the upregulation of GLI1 and GLI2 protein level brought by bFGF as effectively as AZD4547 (Figure 4A). Similarly, FGFR1 KD led to down-regulation of GLI1 and GLI2 in H520 and H1581 cells (Figure 4B). At the transcriptional level, the 24 h bFGF treatment increased GLI2 mRNA levels significantly in H520 (p<0.05) and H1581 (p<0.05) cells, and this increase could be blocked by AZD4547 (Figure 4C). FGFR1 KD decreased mRNA level of GLI2 in H520 (p<0.05) and H1581 (p<0.01) cells (Figure 4D). Immuno-fluorescence staining confirmed that bFGF stimulated the expression of GLI2, and this effect could also be blocked by AZD4547 (Figure 4E).

It is well known that GLI2 was a key transcriptional factor in classical Sonic Hedgehog pathway. However, previous studies showed that the proliferation of lung cancer cell lines H520 and A549 could not be affected by exogenous shh [28] or SMO inhibitor (GDC0449) but could be inhibited by GLI protein inhibitor (GANT61) [21]. These results inspired us to explore whether FGFR1 stimulated GLI2 expression through the classical Sonic Hedgehog pathway. First, treatment with shh for 24 h did not increase the expression of GLI2 either in H520 or H1581 cells (Supplementary Figure S3A), in line with the results of GLI1 expression reported in previous study [28]. Then we also found that the upregulation of GLI2 caused by bFGF treatment could not be blocked by SMO inhibitor GDC0449 (Supplementary Figure S3B). Furthermore, shh treatment could not accelerate the growth of H520 and H1581 oncospheres. Importantly, the addition of shh failed to rescue the effect of FGFR1 KD (Supplementary Figure S3C).

As AZD4547 is a multitargeted inhibitor against FGFR1/2/3, it is critical to evaluate the role of other FGFRs besides FGFR1 in our study. Based on our RNA-seq data, compared with the paired paratumor tissues, mRNA expression level of FGFR1 was higher in LSCC tissues (p<0.01). However, the mRNA expression of FGFR2 and FGFR3 was comparable between tumor and paired normal tissue (Supplementary Figure S4A). Moreover, FGFR1 was the sole gene found to be amplified in LSCC tissue based on our own data (37.8%) and TCGA (20.8%) (Supplementary Figure S4B). The relative mRNA and protein levels of FGFR1/2/3 were also examined in H520 and H1581 cells. FGFR1 was highly expressed in both cells; FGFR2 was only hyperexpressed in H1581 cell; FGFR3 mRNA level was much lower in both cell lines compared with FGFR1 and FGFR2 (Supplementary Figure S4C and S4D): these data are in line with the results described by Malchers F et al. in their studies [19]. We then chose H1581 to explore the relationship between FGFR2 and GLI2. Knockdown of FGFR2 with specific siRNA failed to affect the expression of GLI2 at all (Supplementary Figure S4E). These results seemed to rule out the involvement of FGFR2/3 in regulating GLI2 in FGFR1-amplified lung cancer cells, especially in LSCC.

Our findings suggested that FGFR1 signaling directly regulates GLI2 expression in FGFR1-amplified cells through the ERK pathway but not through the classical Sonic Hedgehog pathway.

### GLI2 is required for maintaining the stem-like phenotype of FGFR1 -amplified cells

**Figure 5:**
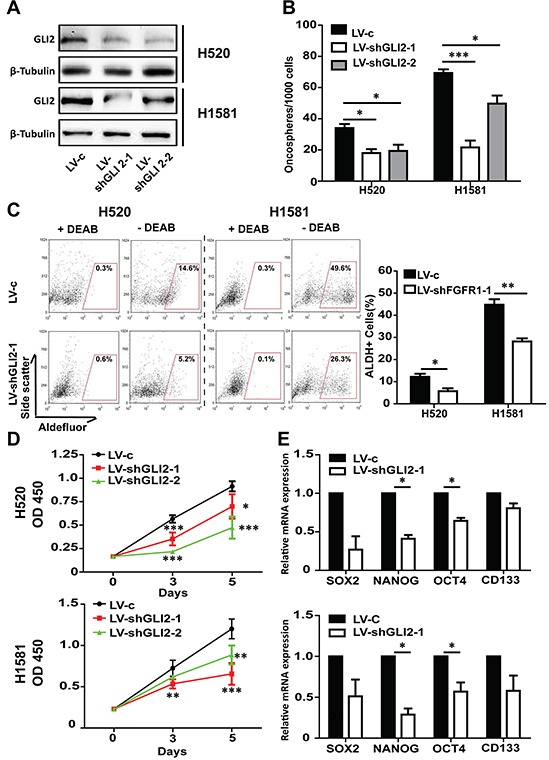
GLI2 is required for FGFR1-amplified lung cancer cell growth and maintenance of self-renewal in oncospheres **A.** Western blot analysis of GLI2 levels in H520 and H1581 cells that were infected with control lentivirus (LV-c), or shRNA targeting GLI2 (LV-shGLI2-1 and LV-shGLI2-2). **B.** Oncosphere formation assay in H520 and H1581 cells that were infected with either LV-c, LV-shGLI2-1 orLV-shGLI2-2 (Error bars represent SEM; N=6, *p<0.05, **p<0.01, and *** p<0.001 Student's t test). **C.** ALDH activity assay in H520 and H1581 cells that were infected with either LV-c or LV-shGLI2-1. (Error bars represent SEM, N=3; *p<0.05 and ** p<0.01, Student's t test). **D.** CCK-8 assay of H520 and H1581 cells that were infected with LV-c, LV-shGLI2-1 or LV-shGLI2-2. (Error bars represent SD; N=6, *p<0.05, ** p<0.01, and ***p<0.001, two-way ANOVA, followed by post-hoc tests). **E.** The relative mRNA levels of stem cell markers CD133, NANOG, OCT4 and SOX2 in H520 and H1581 cells that were infected with either LV-c or LV-shGLI2-1.(Error bars represent SEM, N=3;*p<0.05, Student's t test).

We further assessed the effect of genetic inhibition of GLI2 on the stem-like phenotype of lung cancer cells. Western blot and immunofluorescence experiments confirmed the GLI2 silencing effect (Figure 5A and Supplementary Figure S5). GLI2 KD produced the similar effect as in FGFR1 inhibition. Briefly, GLI2 KD slowed the growth and the oncosphere formation ability of H520 and H1581 (Figure 5B and 5D). And the proportion of ALDH positive cells reduced dramatically in GLI2 KD cells compared with vector control in H520 (from 14.6% to 5.2%) (p<0.05) and in H1581 (from 49.6% to 26.3%) (p<0.01) (Figure 5C). And compared with the vector control, GLI2 knockdown inhibited the expression of stem cell markers in H520 and H1581 (Figure 5E). GLI2 KD decreased mRNA level of GLI1 in H520 (p<0.01) and H1581 (p<0.01) cells (Supplementary Figure S6).

**Figure 6:**
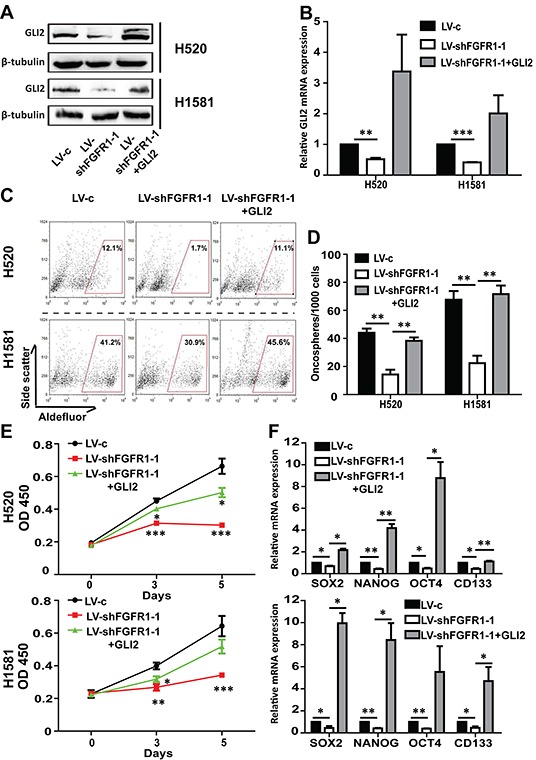
The effect of FGFR1 knockdown can be rescued by GLI2 expression **A.** Western blot and **B.** qPCR analysis showed GLI2 expression levels in H520 and H1581 cells that were treated with LV-c, LV-shFGFR1-1 or LV-shFGFR1-1+GLI2 plasmid. FGFR1 knockdown H520 or H1581 cells were transiently transduced with plasmid expressing wild-type GLI2 or control vector. (Error bars represent SEM; N=3, *p<0.05, **p<0.01, and ***p<0.001 Student's t test) **C.** ALDH activity assay in H520 and H1581 cells that were treated with LV-c, LV-shFGFR1-1 or LV-shFGFR1-1+GLI2 plasmid. **D.** Oncosphere formation assay for H520 and H1581 cells that were treated with LV-c, LV-shFGFR1-1 orLV-shFGFR1-1+GLI2 plasmid (Error bars represent SEM; N=6, **p<0.01, Student's t test). **E.** CCK-8 assay of H520 and H1581 cells that were treated with LV-c, LV-shFGFR1-1 or LV-shFGFR1-1+GLI2 plasmid. (Error bars represent SD; N=6, *p<0.05, **p<0.01, and *** p<0.001, two-way ANOVA, followed by post-hoc tests). **F.** The relative mRNA levels of stem cell markers CD133, NANOG, OCT4 and SOX2 in H520 and H1581 cells that were treated with LV-c, LV-shFGFR1-1 orLV-shFGFR1-1+GLI2 plasmid. (Error bars represent SEM, N=3 *p<0.05, and **p<0.01 Student's t test).

We then transiently transfected the FGFR1 KD cell lines with GLI2 plasmid. GLI2 overexpression was confirmed by Western blot and qPCR experiments (Figure 6A and 6B). The inhibited growth of FGFR1 KD oncospheres could be liberated after GLI2 plasmid transfection (Figure 6D and 6E). And the decrease of ALDH positive ratio and the stemness gene expression caused by FGFR1 KD could also be rescued by re-expression of GLI2 in FGFR1 KD cells (Figure 6C and 6F).

Together, these results showed that GLI2 was involved in FGFR1-dependent Hedgehog signaling pathway, which was critical for maintaining the stem-like phenotype of FGFR1 -amplified lung cancer cells.

### Higher expression of FGFR1 and GLI2 correlate with shorter progression free survival in NSCLC patients

**Figure 7:**
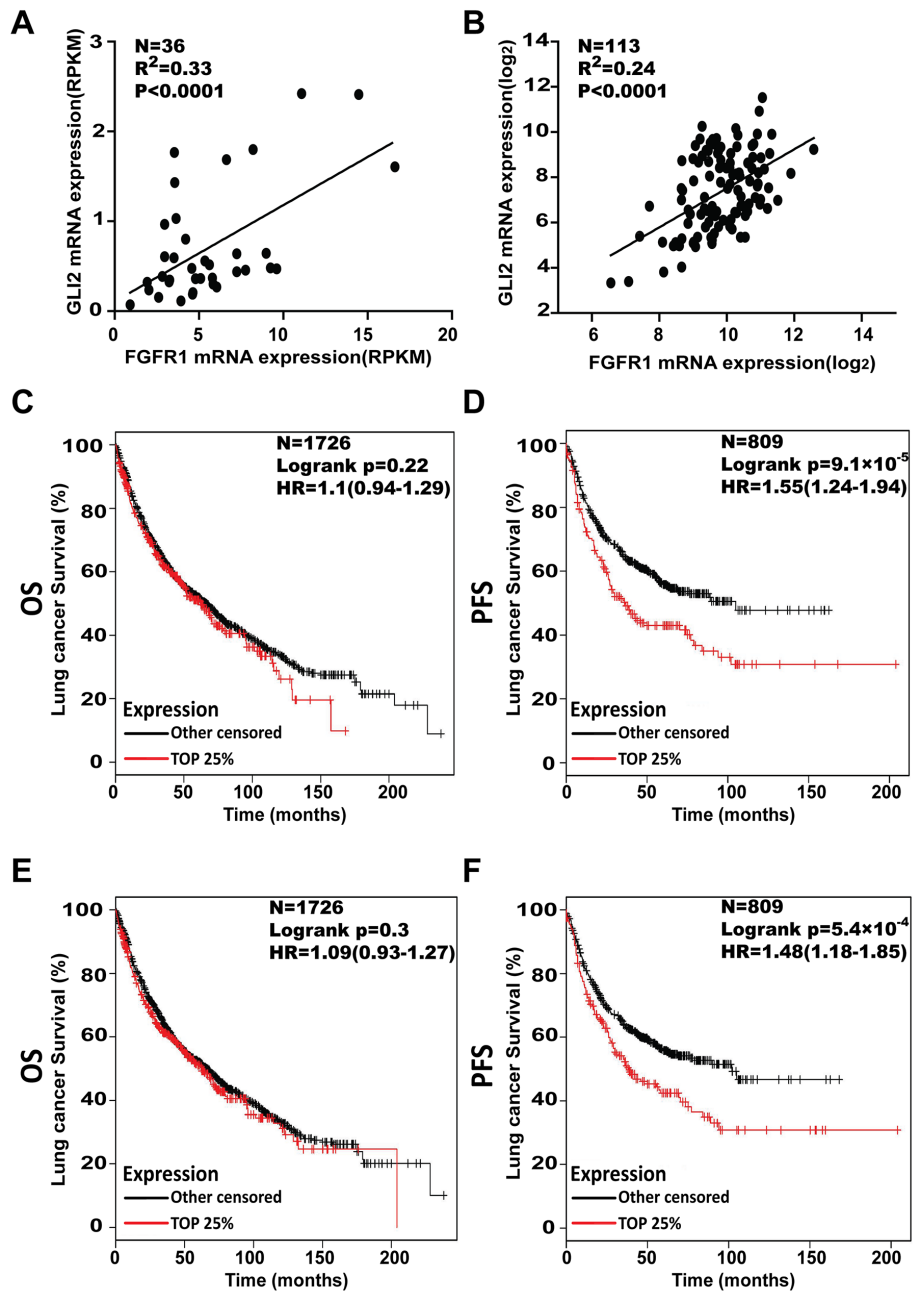
Higher levels of FGFR1 and GLI2 both associate with shorter progression free survival in NSCLC patients **A.** Linear correlation analysis of FGFR1 with GLI2 expression, as measured by RNA-seq, and the data was labeled as Reads Per Kilo-bases per Million-reads (RPKM) in the LSCC tissues (N=36). Each sample is represented by a dot. The extent of the Pearson correlation is indicated by R^2^. **B.** Linear correlation analysis of FGFR1 with GLI2 expression. Data were from TCGA sample (N=113). Each sample is represented by a dot. The extent of the Pearson correlation is indicated by R^2^. **C.** The effect of FGFR1mRNA expression level on the overall survival OS; N=1726) and **D.** progression free survival (PFS; N=809) in lung cancer patients was analyzed and the Kaplan-Meier plots were generated by Kaplan-Meier Plotter (http://www.kmplot.com). The upper quartile were compared with lower FGFR1 patients. **E.** The effect of GLI2 mRNA expression level on the overall survival (N=1726) and **F.** progression free survival (N=809) in lung cancer patients was analyzed and the Kaplan-Meier plots were generated by Kaplan-Meier Plotter (http://www.kmplot.com). The upper quartile was compared with the lower GLI2 patients.

Given the functional link between FGFR1 and GLI2, we assessed whether these two genes might be co-expressed in LSCC tumors. Based on our own RNA-seq data in LSCC patients, we found that there was a positive correlation between FGFR1 and GLI2 (N=36, p<0.0001, R^2^=0.33) (Figure 7A), these results were confirmed in data from The Cancer Genome Atlas (TCGA) (N=113, p<0.0001, R^2^=0.24) (Figure 7B). To further explore the clinical significance of FGFR1/GLI2 signaling.

We then tested the relationship between the FGFR1 or GLI2 expression level and the survival of NSCLC patients, 2437 lung cancer samples from publicly available datasets (2015 version) (http://kmplot.com/analysis/index.php?p=servicecancer=lung) were used for analysis [29]. The Kaplan-Meier analyses demonstrated that the higher expression of FGFR1 was correlated with shorter progress free survival (PFS) (N=809, p=9.1×10^−5^, univariate Cox, in Figure 7D). A similar negative connection was also found between the GLI2 expression level and PFS (N=809, p=5.4×10^−4^, univariate Cox in Figure 7F). However from these datasets we failed to establish a statistically significant connection between FGFR1 or GLI2 expression and overall survival (OS) (for FGFR1, N=1726, p=0.22, univariate Cox in Figure 7C; or for GLI2, N=1726, p=0.3, univariate Cox in Figure 7E).

## DISCUSSION

**Figure 8:**
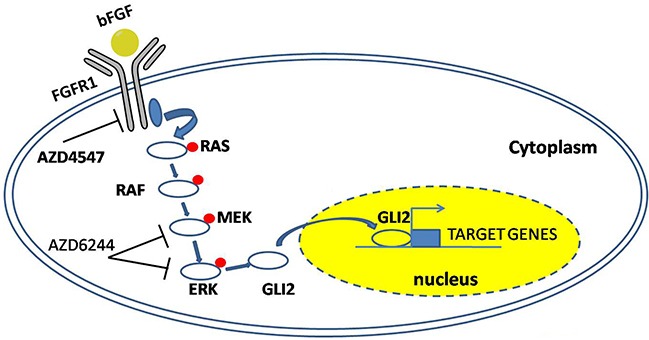
A model summarizing the FGFR1-ERK-GLI2 signaling axis that regulates the stem cell-like phenotype in NSCLC cells After binding with the FGFR1, bFGF stimulates the phosphorylation of FGFR1 and its downstream signaling component, leading to the upregulation of GLI2 expression and its transcriptional activity.

Over the past few years, accumulating evidences have suggested the subtype of cells with stem cell-like phenotype was the source of resistance and relapse. Indeed, traditional cancer drugs had been developed to inhibit differentiated cancer cells, and failed to eradicate stem cell-like cells was thought to be a critical factor of recurrence. Herein the results from the ALDH activity, sensitivity against FGFR1 inhibitor/knockdown, and the expression of stem cell markers, supported the notion that FGFR1 promoted the stem cell-like phenotype of FGFR1-amplified LSCC. The survival and accumulation of these stem cell-like cells (possibly with elevated FGFR1/GLI2) might explain in the clinic the association with shorter PFS. As elimination of cancer stem cell-like cells provided an effective strategy to overcome tumor resistance and reduce relapse, our findings in this study shed some light on the molecular mechanisms of the molecular mechanisms of maintaining the stem cell-like phenotype, in which FGFR1 regulated the GLI2 expression through ERK pathway (Figure 8), and provided a novel target for the treatment of a subtype of NSCLC cells.

Aberrant FGFRs signaling had been implicated in a diverse spectrum of human cancers, including those in the lung, breast, and stomach [30]. The mechanism of FGF/FGFR signaling activation varies in different cancers, resulting from gene amplification, mutation, trans -location or SNPs [30]. Among these, FGFR1 amplification is a prominent gene alteration in LSCC. The incidence of FGFR1 amplification has been reported to be approximately 22% in LSCC, but only 3.4% in adenocarcinoma [11, 31]. A relative higher rate was reported in East Asian patients (31%) [32]. It was reported that suppression of FGFR1 by FGFR inhibitor or shRNA can repress the proliferation of FGFR1-amplified cells, such as H1581 and DMS114 [19]. In this study, we discovered that FGFR1 inhibitor AZD4547 decreased the ALDH-positive population, which was regarded as a stem cell marker in FGFR1-amplified lung cancer cells. Furthermore, AZD4547 inhibited the growth of oncospheres both in H520 and H1581 at low concentrations, these results were in line with the effect of FGFR1 knockdown. In the xenograft model of H1581 cells, we confirmed that the FGFR1 inhibitor AZD4547 could repress the growth of tumor inoculated either from the oncospheres or parental cells; the inhibitor could also reduce the ALDH positive proportion of tumors in both these two models. These results seemed to implicate that for stem-cell enriched LSCC cells (especially when they develop treatment-resistance), inclusion of FGFR1 inhibitors was a feasible therapy strategy. It was interesting to note that the basal ALDH positive proportions in H1581 cells were different in AZD4547 treatment experiment (Figure 1A) and in the control lentivirus stable transfection experiment (Figure 5C). The difference may attribute to the different culture conditions in these two experiments. In the former study the cells were cultured in low serum medium (1% FBS) for 5 days to be followed by flow-cytometry assay, but the latter were cultured in normal medium (10% FBS). From our observation H1581 cell lines seemed to be more sensitive to the alteration of the serum concentration. Different cell state may lead to different basal ALDH positive level. ALDH positive proportion in the control lentiviral transfected cells was similar to the wildtype cells when they were cultured in normal condition (data not shown). On the contrary, the H520 seemed to be less sensitive to the change of culture condition.

Aberrant Hedgehog signaling has been implicated in a diverse spectrum of human cancers, including lung cancer. Both previous immunohistochemical studies and TCGA data indicated the activation of Hedgehog pathways in LSCC [22, 33]. In particular, overexpression of GLI2 was evident among different database results, and closely connected with classical subtype of LSCC [21]. GLI2 is the primary effector of Hedgehog signaling cascade. Hedgehog signaling activation leads to GLI2 stabilization and then induces the transcriptional upregulation of GLI1 [25]. GLI1 protein without repressor domain is constitutively active, and potentiates the transcription of Hedgehog-GLI2 target genes. And it was also reported in some papers that GLI1 was able to induce GLI2 expression [34]. Because GLI2 and GLI1induce transcription of overlapping but distinct sets of target genes, GLI2-mediated GLI1 forms a positive feedback and leads to augmentation of Hedgehog signaling quantitatively as well as qualitatively [35]. Genetic alterations, including loss of PTCH function, constitutively active SMO, and amplification of GLI1/GLI2 have been reported to activate downstream Hedgehog signaling independent of ligands in various cancers. However these mechanisms were rare in LSCC. Indeed, the mechanisms regulated Hedgehog pathway in LSCC was still uncertain. Moreover, it was reported that GLI function can be modulated in a SMO-independent manner by PI3K/AKT, RAS-MEK signaling [36-38], and the activity of GLI can be activated or suppressed in different models [27, 39-41]. Previously Hedgehog pathway was reported to play an important role in maintaining the stem cell-like phenotype of LSCC [20]. In our study, we uncovered a link between FGFR1 and GLI2: FGFR1 enhanced the expression and transcriptional activity of GLI2 through ERK phosphorylation. In the further study, using FGFR1 KD and GLI2 KD cell lines, we confirmed that FGFR1 inhibition could dramatically repress the expression of GLI2 and the related gene GLI1. In the rescue experiment, we found that overexpression of GLI2 could offset the effect brought by FGFR1 KD in both FGFR1-amplified cell lines. FGFR1or GLI2 was previously regarded as a promising target in LSCC, and we discovered the correlation of FGFR1 and GLI2 in LSCC patients. To our knowledge, this provided initial evidence to connect the FGFR1 and Hedgehog signaling pathways, and explain how FGFR1 signaling regulates stem cell properties in LSCC. FGFR1/GLI2's involvement in the modulation of self-renewal program of the stem cell-like cells were further supported by analysis of these factors in the large cohorts of NSCLC patients (over 2437 cases), representing an integration of data from multiple published studies. As we suggested in the study that higher FGFR1 or GLI2 levels were correlated with shorter PFS, it is worth considering these two pathways together in future clinical studies for the treatment of LSCC.

Herein for the first time we demonstrated that FGFR1 promoted stem cell-like phenotype in lung cancer cells harboring FGFR1 amplification, especially in LSCC, partly through stimulating the expression and activity of GLI2. Furthermore, our results revealed a functional interaction between FGFR1 and Hedgehog/GLI2 pathway. FGFR1 and GLI2 may cooperate in driving growth and maintenance of LSCC stem cell-like cells. These provide a rational strategy of combination of FGFR1 and GLI inhibitors for the treatment of FGFR1-amplified LSCC tumors in the future.

## MATERIALS AND METHODS

### Cell cultures and reagents

Human lung squamous cancer cell line H520 and large cell line H1581 cells were purchased from American Type Culture Collection (ATCC; Manassas, VA, USA). Cells were routinely cultured in RPMI 1640 (Hyclone) supplemented with 10% fetal bovine serum (Gibco), 100 U/ml penicillin and 100 μg/ml streptomycin. All cells lines were grown in 5% CO_2_ atmosphere at 37°C. FGFR1 inhibitor AZD4547 was kindly provided by *AstraZeneca Pharmaceutical Co.*. AZD6244 and GDC0449was purchased from Selleck Chemicals.

### Lentiviral RNAi constructs, cell transduction, plasmids

The lentivirus labeled with RFP against FGFR1 and GLI2 were purchased from HanBio (www.hanbio.net). The specific target sequences of FGFR1 and GLI2 were listed in the Supplementary Table S2. Stable cell lines were generated by infection of cells with lentivirus, which was carried out in 24-well plate with serum-free DMEM medium. H520 and H1581 cells were transduced with lenti-sh-FGFR1 and lenti-sh-GLI2 virus at the infection MOI ≥90 at 37°C with 8μg/ml polybrene for 24 h. Then culture medium with 10% FBS was replaced and cells were continuously cultured for 4 to 6 days followed by selection with G418 (Invitrogen) at 500 μg/ml. pCS2-MT GLI2 FL was a gift from Erich Roessler (Addgene plasmid # 17648)[42]. siRNA was purchased from GenePharma (Shanghai, China)

### Oncosphere cell culture

Oncosphere cell culture was performed according to the published protocol with some modifications [2, 3, 20]. Briefly, oncospheres were enriched from H520 and H1581 cells by culturing 10,000 cells/ml of CSC culture medium [serum-free DMEM-F12 medium (Life Technologies, Grand Island, NY) containing B-27 Supplement (Life Technologies, Grand Island, NY), 20 ng/ml basic fibroblast growth factor (Gibco), and 20 ng/ml epidermal growth factor (Gibco)] in ultra-low attachment flasks (Corning, Corning, NY) that support the growth of undifferentiated oncospheres.

### CCK-8 assay

The effect of AZD4547 on the viabilities of H520 and H1581 oncospheres cells were examined by CCK-8 assay kit (Dojindo Laboratories, Kumamoto, Japan), according to the previous study with some modification [20]. Briefly, the oncospheres were dissociated into single-cell suspensions by accutase (Stem Cell Technologies). Then the cells were seeded in 96-well plates at a density of 3,000 cells per well (N=6) in the presence or absence of AZD4547 with different concentrations. After 96 h, 10 μl of CCK8 solution was added to each well and incubated for 4–6 h at 37°C. The absorbance at 450nm was measured by the microplate reader (Synergy2, BioTek, Winooski, VT). The IC50 value was calculated in GraphPad Prism software. For the proliferation assay, cells with different treatment were seeded in 96-well plates at a destiny of 1000 cells/well (N=6). At the assigned time point, the cells were obtained and the OD value at 450nm was measured.

### Sphere formation assay

Sphere formation assay was performed according to Dontu's description with some modification [43]. Briefly, Adherent H520 and H1581 cells were gently trypsinized, washed, and then seeded (3000/well) in 6-well ultra-low attachment plates. Spheres (>75 μm diameter) were counted after 10–14 days with or without FGFR1 inhibitor AZD4547 (1 μM). To assay effects of RNAi knockdown of FGFR1, GLI2, H520 and H1581 were stable transfected with LV-c, LV-shFGFR1-1/2, and LV-shGLI2-1/2 lentivirus and led to form oncospheres. In the rescue experiment, the cells lines stable transfected with LV-shFGFR1-1 lentivirus and then transient transfected with plasmid pCS2-MT GLI2 FL and its relevant control vector and led to form oncospheres. The medium was refreshed twice a week, and the numbers of oncospheres were determined using Leica digital camera.

### Flow cytometry analysis

The Aldefluor Assay Kit (Stem Cell Technologies) was used to determine the ALDH positive cells. The assay was performed according to manufacturer's instructions with modifications [44]. Cell suspensions were counted and suspended in Aldefluor assay buffer, which was divided into two groups. The baseline fluorescence was tested by pretreatment with ALDH-specific inhibitor diethylaminobenzaldehyde (DEAB) for 10 minutes before incubation with ALDH enzyme substrate bodipy-aminoacetaldehyde (BAA) for 45 minutes at 37°C. For the analysis of ALDH-positive cells, DEAB-treated sample was used as a negative control and ALDH activity in the presence of DEAB was considered as a baseline. Cells were analyzed on a FACS Calibur (BD Biosciences) flow cytometry.

### Quantitative real-time PCR (qRT-PCR) and western blot analysis

Total RNA was extracted from cells with Trizol reagent (TaKaRa, Dalian, China) to the manufacturer's protocol. PrimerScript reverse transcriptase (RT) reagent kit (TaKaRa) was used to synthesize cDNA from total RNA. Quantitative real-time PCR was performed on ABI 7900HT by using SYBR Premix Ex Taq (TaKaRa). Western blot was performed to detect the changes of protein levels under different treatments. Cell samples were lysed in lysis buffer (Thermo Scientific, Rockford, IL, USA) containing Complete Protease Inhibitor Cocktail, Phosphatase Inhibitor Cocktail and 2 mM phenylmethylsulfonyl fluoride (PMSF). The lysates were collected. Immunoblotting was carried out as previously described following the methods as described before [45]. The detailed procedure, primers for qRT-PCR (Supplementary Table S1) and antibodies used in western blot are described in the Supplementary Materials and Methods.

### Immunofluorescence microscopy

Cells grown in chamber wells were fixed in 4% paraformaldehyde for 10 minutes at room temperature then rinsed with PBS for 5 minutes followed by permeablizing in 0.5% Triton X-100 for 10 minutes and blocking in 1% bovine serum albumin in PBS for 30 minutes at room temperature with shaking. Cells were incubated with GLI2 antibody at 1:200 dilutions (Epitomics) overnight at 4°C. Alternatively, cells were incubated with isotype controls, including rabbit IgG conjugated with Alexa Fluor 488 and 594 (Cell Signaling, 2975 and 8760). Cells were rinsed in PBS for 5 minutes, once with PBS and mounted with Prolong anti-fade with DAPI (Life Technologies). The labeled cells were visualized under a microscope (Leica DFC420C) and images were processed using Adobe Photoshop software.

### Analysis of publicly available datasets

To analyze the effect of *FGFR1* or *GLI2* expression on prognostic of lung cancer patients, we generated Kaplan-Meier survival curve of NSCLC patients with low or high expression of *FGFR1 or GLI2* by using Kaplan-Meier Plotter (www.kmplot.com/analysis). [29, 46].

Data from TCGA were analyzed using cBIO software (http://www.cbioportal.org/public-portal/) software to correlate gene expression of “FGFR1” and “GLI2” in 119 human LSCC. Then the data of FGFR1 and GLI2 were downloaded and the coorelationship were analyzed in Graphpad software. [47, 48]

### Statistical analysis

The GraphPad Prism software (GraphPad Software Inc., La Jolla, CA, USA) was used in data processing and statistical analysis of significance. All data were presented as means±SEM or SD where indicated formats least three replicate experiments. Comparisons between two groups were performed using Student's t tests and ANOVA with Tukey post-hoc test was used to compare three or more groups, p<0.05 was considered significant.

## SUPPLEMENTARY MATERIALS FIGURES AND TABLES


