# Supplementary material for: FGFR1 promotes the stem cell-like phenotype of FGFR1-amplified non-small cell lung cancer cells through the Hedgehog pathway
Source: Oncotarget. 2016 Feb 25;7(12):15118–34. doi: 10.18632/oncotarget.7701 (PMC4924774; doi:10.18632/oncotarget.7701)
Supplement: Supplementary file 1 [file oncotarget-07-15118-s001.pdf]

SUPPLEMENTARY MATERIALS AND METHODS

Tissue acquisition

The collection of human samples and the protocols for this study were approved by the Institutional Ethics Committee of Shanghai Jiao Tong University. All patient-derived samples were collected with informed consents from individuals received surgery as the primary treatment at the Shanghai Chest Hospital between April 2009 and December 2012. Tumor samples were collected immediately following surgical resection, and then kept in pre-cold RPMI-1640 medium with 5% FBS and 1 × Penicillin / Streptomycin or in Histidine-Tryptophan-Ketoglutarate tissue preservation solution if the estimated shipping time was longer than one hour. All samples were de-identified by the National Tumor Tissue Bank of China before further experiments. Samples were anonymized, and sectioned for further analysis.

Detection of copy-number alterations and *Whole Transcriptome Sequencing (RNA-seq)*

Copy-number alterations were detected using an in house–developed method called exon-specific copy-number (exon-CNV) estimation from sequencing reads depth. Briefly, exon-CNV follows the following procedures: (i) all exon capture regions were merged together if they have overlap. Then, the high GC content's regions like CpG Islands were excluded for the reason of PCR amplification, capture and sequencing bias; (ii) collection of read depth from tumor and normal samples in merged capture regions and calculating the ratios of read depth tumor VS normal; (iii) constructing the probability density function for these ratios, we can identified the highest peak for an example if highest peak was 1.05 so the tumor overall ploidy was 1.05*2; (iv) normalization according to the contamination of the tumor sample with normal cells and according to the tumor overall ploidy; (v) calculation of all copy numbers for the gene segments were analyzed using Control-FREEC, and copy-number alterations were visualized using Circos tools.

RNAseq was performed following previous study with brief modification [19]. RNA is isolated using Trizol and RNeasy Min Elute Cleanup Kit from tissue samples. Following extraction, the quantity was measured on Nanodrop and the integrity of total RNA was determined using RNA 6000 Nano Kit on Aligent 2100 Bioanalyzer. All the sequencing reads were mapped to the Ensembl GRCh37.62 B (hg19) reference genome using an RNA RNA-seq analysis tools TopHat and Cufflinks for expression estimation base on known set of reference transcripts from Ensembl v.58. The differential expression is calculated by Cuffdiff based on transcript abundances and differentially expressed and labeled as Reads Per Kilo-bases per Million-reads (RPKM).

Quantitative real-time RT-PCR

Total RNA was extracted from cells with Trizol reagent (TaKaRa, Dalian, China)to the manufacturer's protocol. PrimerScript reverse transcriptase (RT) reagent kit (TaKaRa) was used to synthesize cDNA from total RNA. Quantitative real-time PCR was performed on ABI 7900HT by using SYBR Premix Ex Taq (TaKaRa). PCR was performed using the following conditions: denaturing at 95°C for 10 s, followed by 40 cycles of 95°C for 5 s and60°C for 30 s. Data were analyzed by using the comparative threshold cycle (Ct) method, and results were expressed as fold difference normalized to GAPDH.The experiments were performedin triplicate and repeated three times.The sequences of the PCR primers are shown in Supplementary Table S1.

Western blot analysis

Western blot was performed to detect the change of protein levels under different treatments.Cell samples were lysed in lysisbuffer (Thermo Scientific, Rockford, IL, USA) containing Complete Protease Inhibitor Cocktail, Phosphatase Inhibitor Cocktail and 2 mM phenylmethylsulfonyl fluoride (PMSF). The lysates were centrifuged at 12,000 rpm for20 min at 4°C, and the supernatants were collected.Immunoblotting was carried out as previously described following the methods as described before [48].In brief, total protein (30 μg per lane) was separated through a 10 % SDS-PAGE gel and transferred onto nitrocellulose(NC) membranes(Whatman, Piscataway, NJ, USA). The membranes were blocked with 5% nonfat milk at room temperature for 1 h and then incubated overnight at 4 °C with specific primary antibodies. The primary antibodies used were as followings: GLI2, FGFR1, FGFR2 (1:1000, Epitomics, Burlingame, CA, USA); p-AKT/AKT (1:2000, Epitomics, Burlingame, CA, USA); p-FGFR (Try653/ 654), GLI1, p-ERK/ERK, FGFR1 (1:1000, Cell Signaling Technology, Danvers, USA). β-tubulin (1:2000, Sigma). After being washed with Tris-buffered saline containing Tween-20 (TBST) three times, the membranes were incubated with appropriate HRP-conjugated secondary antibodies for 1h. Then, the protein bands were detected by using ECL detection

system (Thermo Scientific). The expression of β-tubulin was used as a loading control. Western blotting reagents and images were captured by using ChemiDoc XRS system (BioRad, Hercules, CA, USA).

In Vivo xenograft assay

The cells from oncospheres and parental cells were trypsinized into single cell suspension and counted. Cell suspensions (5×10^6cells) of H1581 in a total volume of 100μl mixed with matrigel at a 1:1 ratio were injected subcutaneously into the right flanks of 4 week-old male BALB/C nude mice(SLAC, Shanghai). When the size reached more than 0.2 cm^3, mice were randomized into control and treatment groups. In the treatment control group, the mice were treated with 12.5mg/kg/d, and the control was treated with corresponding vehicle.Tumor volume (measured by caliper), animal body weight, and tumor condition were recorded twice weekly for the duration of the study. Tumor volume was calculated as described previously: volume= 0.5×tumor length×tumor $width^2$. Tumors were collected and photographed at 3 weeks after inoculation. All mice were housed in the SPF animal facility of Shanghai Chest Hospital in a pathogen-free environment with controlled temperature and humidity, according to the protocols approved by Institutional Ethics Committee of Shanghai Jiao Tong University.

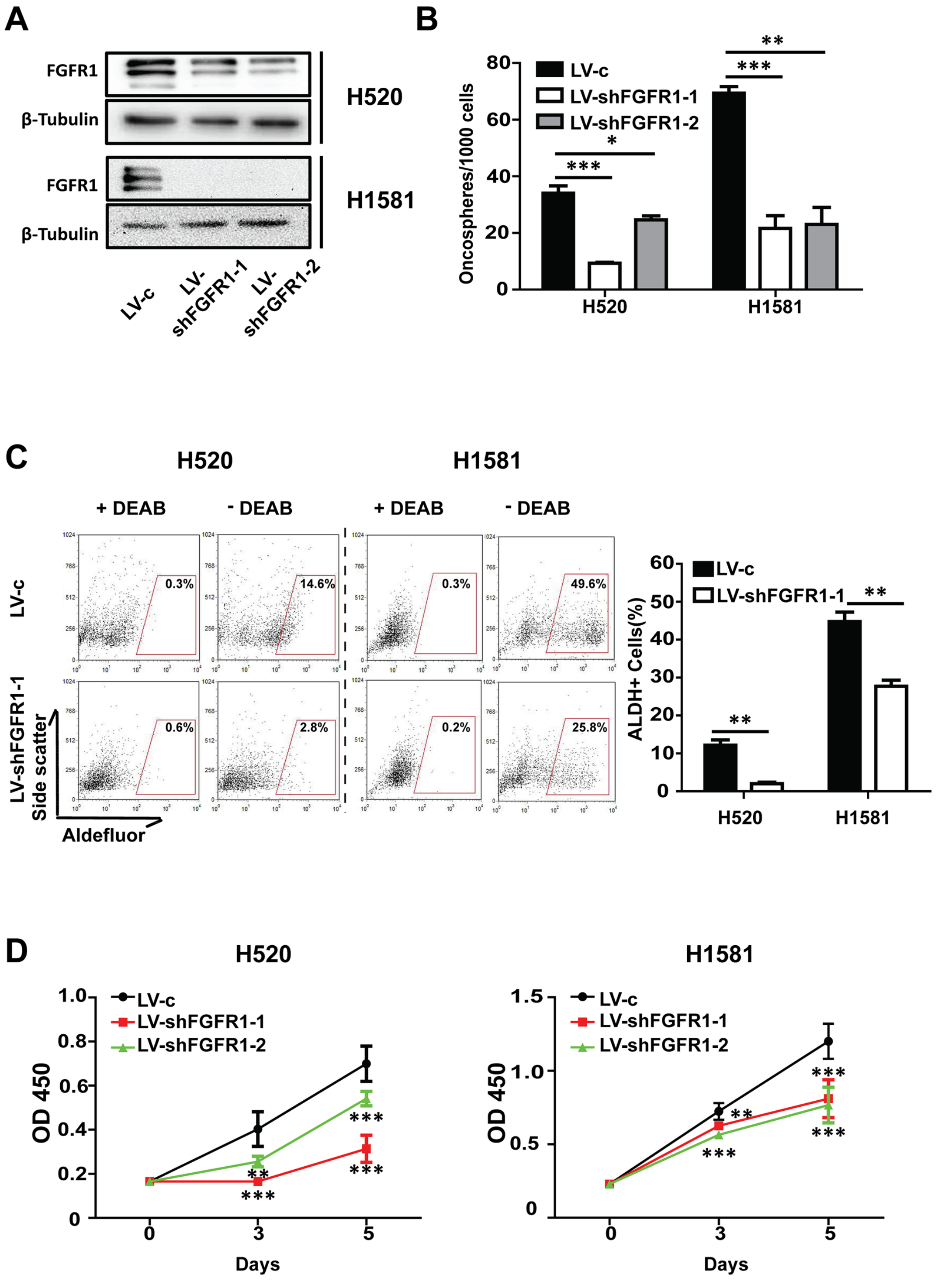

Supplementary Figure S1: FGFR1 knockdown inhibits the growth and self-renewal of oncospheres in H520 and H1581 cells. A. Western blot analysis shows FGFR1 expression levels in H520 and H1581 cells that were infected with LV-c, LV-shFGFR1-1 or LV-shFGFR1-2. **B.** Oncosphere formation assay ofH520 and H1581 cells that were infected with LV-c, LV-shFGFR1-1 or LV-shFGFR1-2. (Error bars represent SEM; N=6, *p<0.05, **p<0.01, and ***p<0.001 Student's t test). **C.** ALDH activity assay of H520 and H1581 cells that were infected with LV-c, LV-shFGFR1-1 or LV-shFGFR1-2(Error bars represent SEM; N=3, **p<0.01 Student's t test). **D.** CCK-8 assay of H520 and H1581 cells that were infected with LV-c, LV-sh FGFR1-1 or LV-shFGFR1-2. (Error bars represent SD; N=6, *p<0.05, **p<0.01, and ***p<0.001, two-way ANOVA, followed by post-hoc tests).

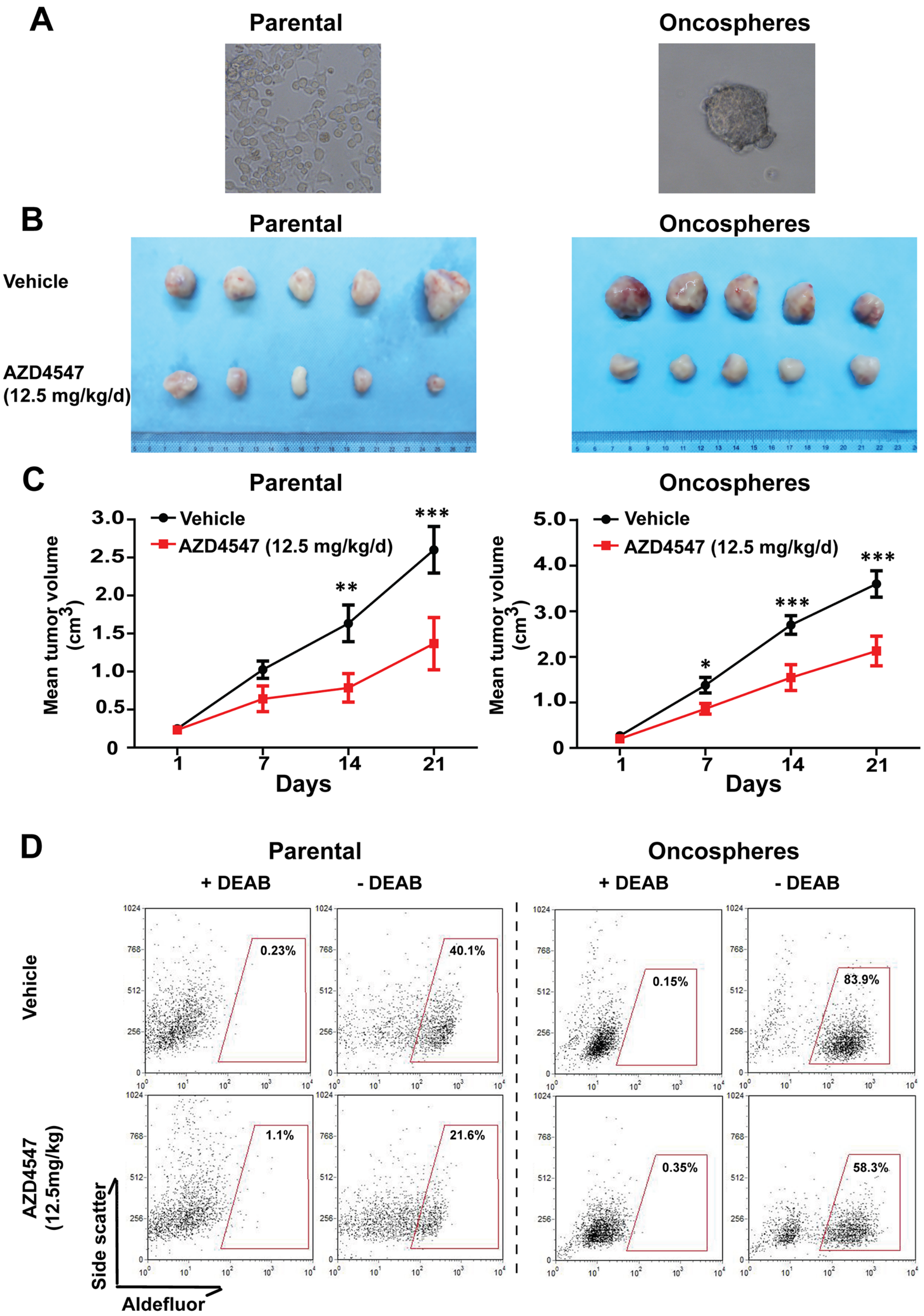

Supplementary Figure S2: AZD4547 inhibits the growth of oncospheres and parental cells of H1581 *in vivo*. A. Representative micrographs of the parental cells and oncospheres of H1581. **B-C.** Growth of xenograft from the parental cells and oncospheres of H1581 treated with vehicle or AZD4547 (12.5 mg/kg/d). **D.** Pooled dissociated tumor cells were assayed for the proportion of ALDH1 positive cells. (Error bars represent SD; N=5, *p<0.05, **p<0.01, and *** p<0.001, two-way ANOVA, followed by post-hoc tests).

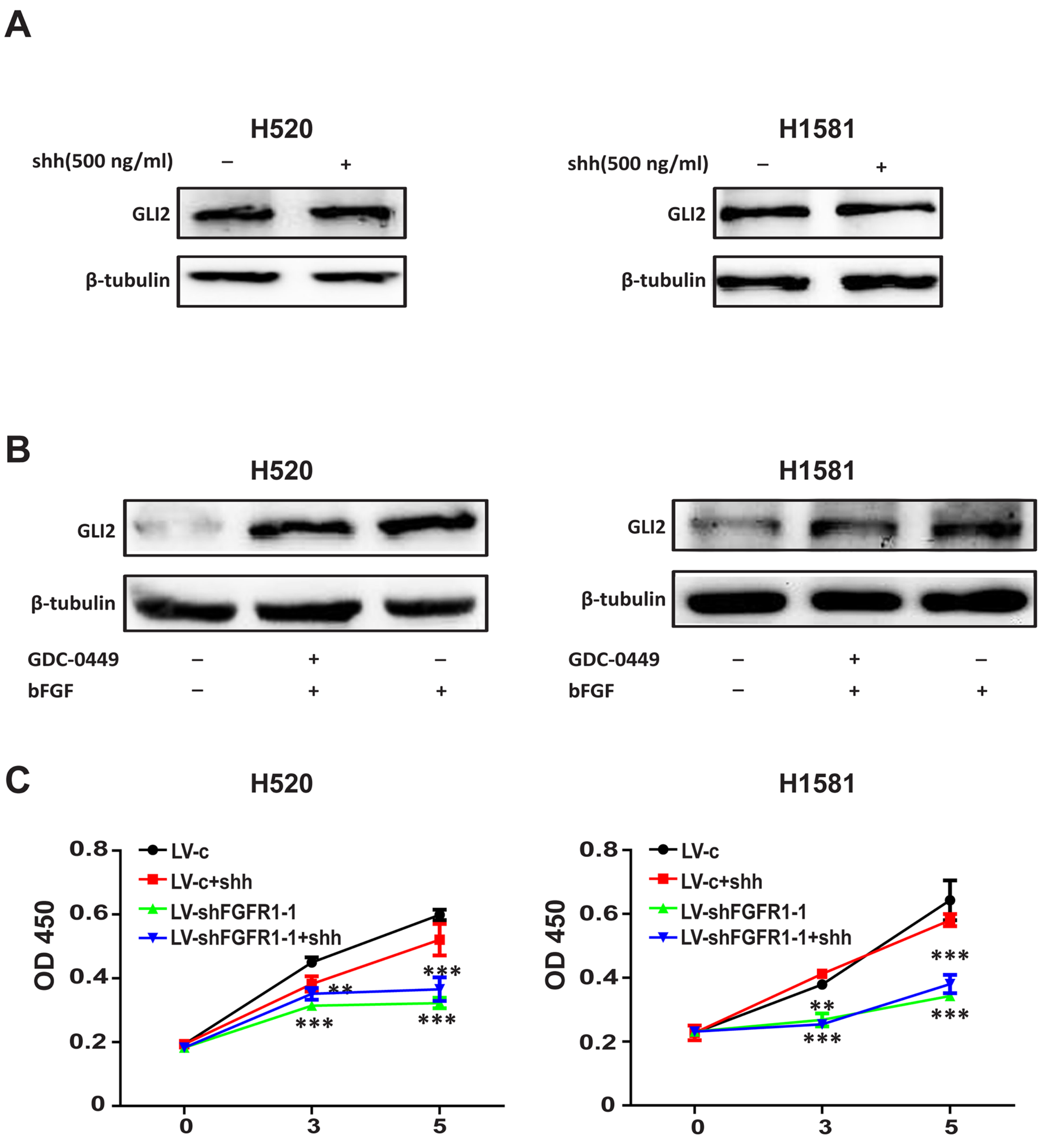

Supplementary Figure S3: GLI2 expression in H520 and H1581 cells is not induced by sonic hedgehog (shh). A. Western blot analysis of GLI2 levels after the treatment of shh (500ng/ml) stimulation for 24h in H520 and H1581 cells. **B.** Western blot analysis of the protein level of GLI2 in H520 and H1581 cells after treatment with bFGF (20 ng/ml) plus heparin (10 µg/ml) or combinations of SMO inhibitor GDC-0449 (10 µM). **C.** CCK-8 assay of H520 and H1581 cells that were infected with LV-c or LV-shFGFR1-1 and in presence of shh (500 ng/ml) or not. (Error bars represent SD; N=6, *$p<0.05$, **$p<0.01$, and ***$p<0.001$, two-way ANOVA, followed by post-hoc tests).

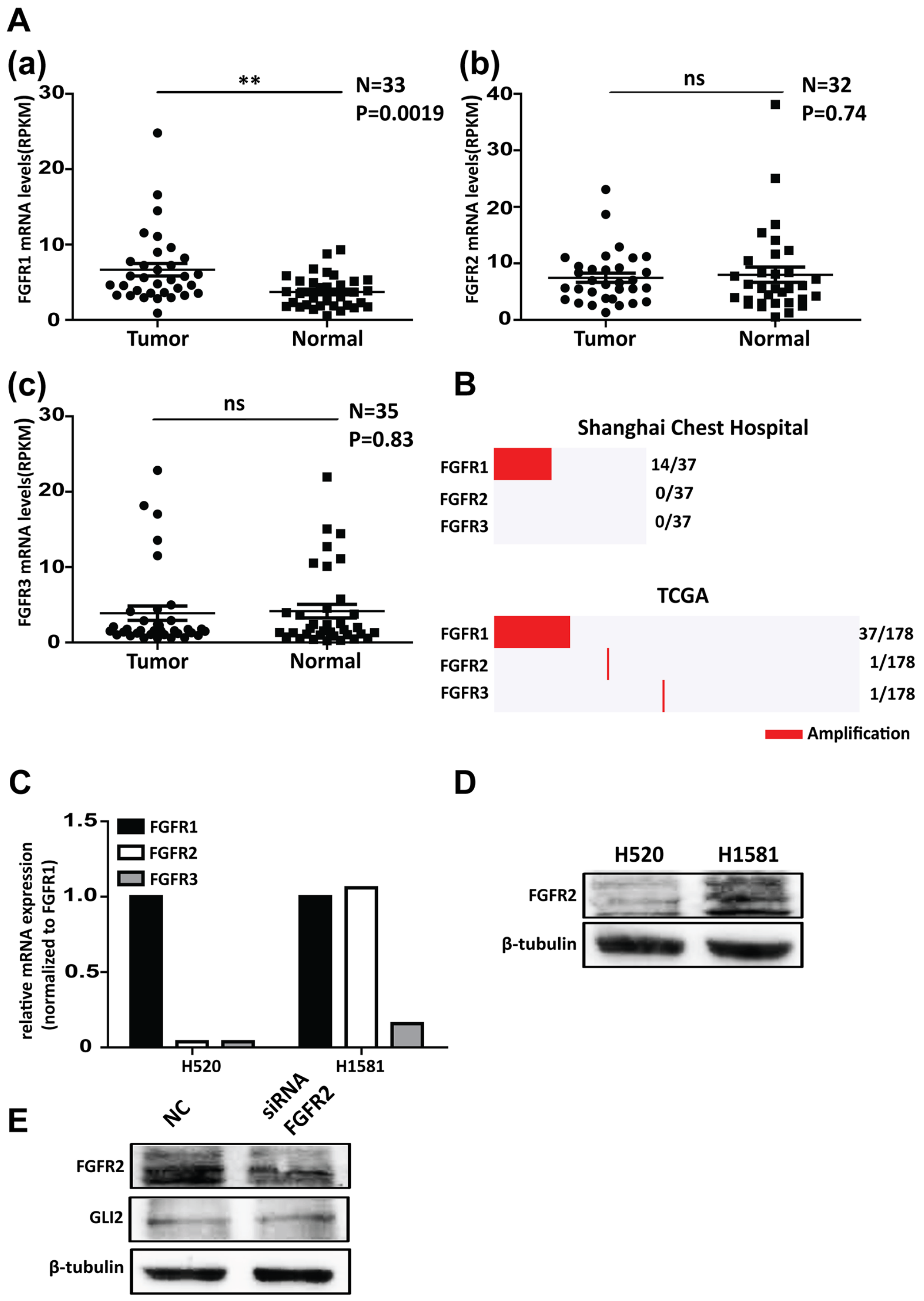

Supplementary Figure S4: FGFRs except FGFR1 do not regulate the expression of GLI2. A. Relative mRNA expression levels of FGFR1 (N=33), FGFR2 (N=32) and FGFR3 (N=35) in LSCC samples and pericarcinous tissue. As measured by RNA-seq, the data was labeled as Reads Per Kilo-bases per Million-reads (RPKM) in the LSCC tissues (Error bars represent SEM; **p < 0.01, Student's t-test). **B.** Copy number variation (CNV) of FGFR1, FGFR2 and FGFR3 in LSCC samples from Shanghai Chest Hospital (N=37) and TCGA data (N=178).The data was edited with HemI 1.0. **C.** Relative mRNA expression of FGFR1, FGFR2, and FGFR3 in H520 and H1581 cells. The mRNA expression of FGFR2 and FGFR3 were normalized to FGFR1. **D.** Western blot analysis of FGFR2 level in H520 and H1581 cells. **E.** Western blot analysis of GLI2 level after knockdown of FGFR2 with siRNA in H1581 cell.

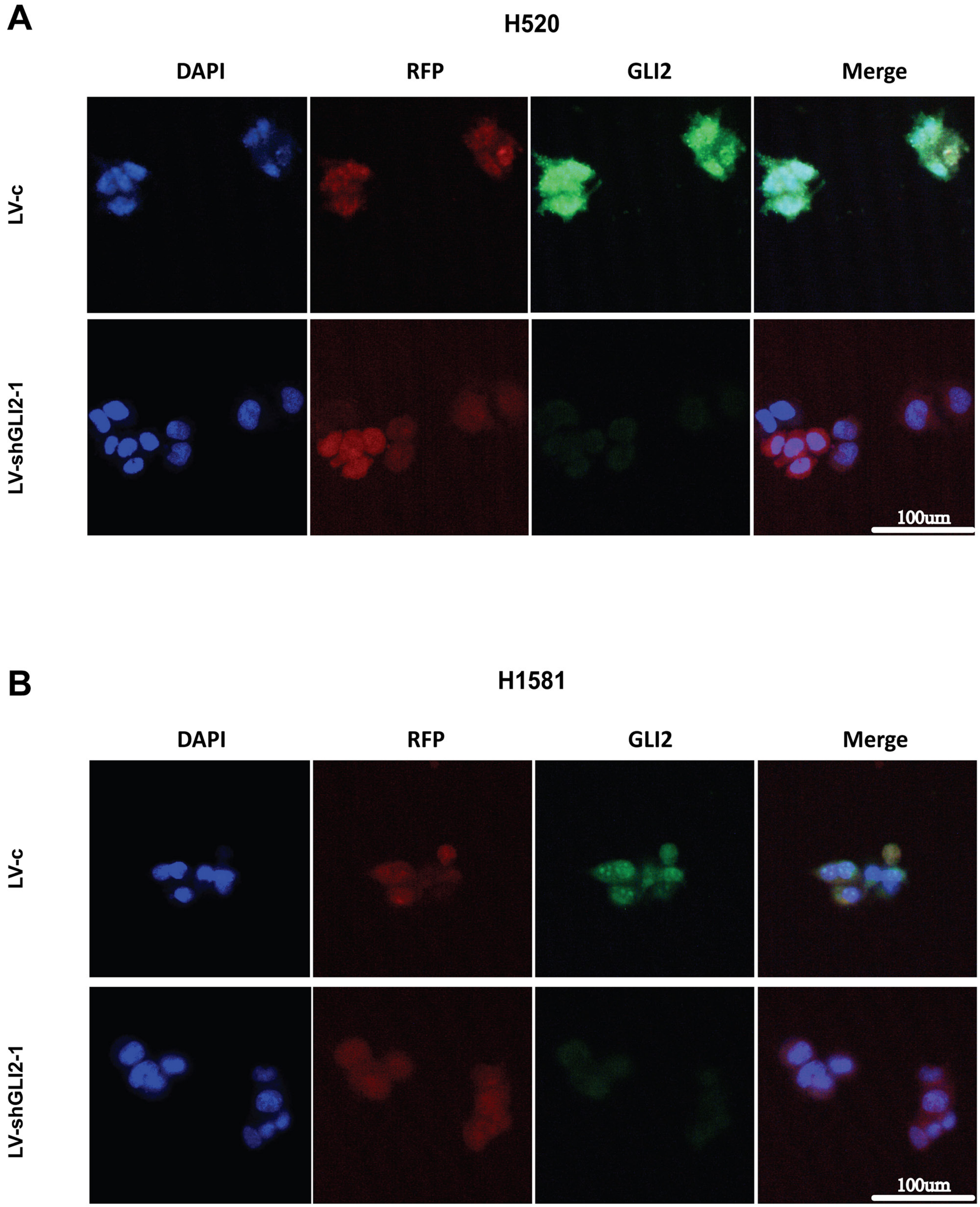

Supplementary Figure S5: Immunofluorescence microscopic analysis of GLI2 expression in H520 and H1581 cells stably transfected with control lentivirus (LV-c) or LV-shGLI2-1. Note the lentivirus contained RFP. Nucleus (blue), RFP (red), GLI2 (green).Scale bar = 100 μm.

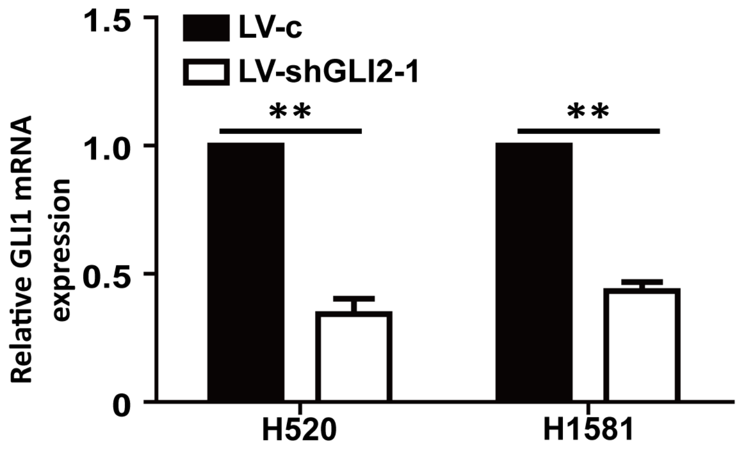

Supplementary Figure S6: GLI2 knockdown repressed the mRNA expression of GLI1. The relative mRNA level of GLI1 in H520 and H1581 cells that were infected with control lentivirus (LV-c) or shRNA targeting GLI2 (LV-shGLI2-1) (Error bars represent SEM; N=3, **p<0.01, Student's t test).

Supplementary Table S1: Primer sequences used in this study

Gene	Sense (5'-3')	Antisense (5'-3')
**OCT4**	GCAATTTGCCAAGCTCCTGAA	GCAGATGGTCGTTTGGCTGA
**NANOG**	CCTGTGATTTGTGGGCCTG	GACAGTCTCCGTGTGAGGCAT
**SOX2**	GTATCAGGAGTTGTCAAGGC	AGTCCTAGTCTTAAAGAGG
**CD133**	TCCACAGAAATTTACCTACATTGG	CAGCAGAGAGCAGATGACCA
**BCL2**	TGGACAACCATGACCTTGGACAATCA	TCCATCCTCCACCAGTGTTCCCATC
**GLI1**	CCAGCCAGAGAGACCAACAG	GTGCGGATAACCGTCTGCAG
**GLI2**	GTATCAGGAGTTGTCAAGGC	AGTCCTAGTCTTAAAGAGG
**PTCH1**	GCCGTGCCCGTGGTCAT	CCCATTGAGAACGCCGAGAT
**HHIP**	GCGGATGAGTTTTGCTTTTA	AGCCATCCCCACTATGC
**FGFR1**	TAATGGACTCTGTGGTGCCCTC	ATGTGTGGTTGATGCTGCCG
**FGFR2**	GGTGGCTGAAAAACGGGAAG	AGATGGGACCACACTTTCCATA
**FGFR3**	CCCAAATGGGAGCTGTCTCG	CCCGGTCCTTGTCAATGCC
**GAPDH**	AGAAGGCTGGGGCTCATTTG	AGGGGCCATCCACAGTCTTC

Supplementary Table S2: shRNA target sequences

Target genes	Sequences
**shFGFR1-1**	5'-CCACAGAATTGGAGGCTACAA-3'
**shFGFR1-2**	5'-GGGTCGGTCATCGTCTACAAGATGA-3'
**shGLI2-1**	5'-GCAACAAAGCCTTCTCCAACG-3'
**shGLI2-1**	5'-CAAGTCACTCAAGGATTCCTGCTCA-3'
